# A systematic review of pharmacogenetic testing to guide antipsychotic treatment

**DOI:** 10.1038/s44220-024-00240-2

**Published:** 2024-04-17

**Authors:** Noushin Saadullah Khani, Georgie Hudson, Georgina Mills, Soumita Ramesh, Lauren Varney, Marius Cotic, Rosemary Abidoph, Alvin Richards-Belle, Lorena Carrascal-Laso, Manuel Franco-Martin, Benjamin Skov Kaas-Hansen, Gesche Jürgens, Barbara Barrett, Huajie Jin, Elvira Bramon

**Affiliations:** 1 Mental Health Neuroscience Research Department, Division of Psychiatry, University College London, London, UK; 2 Department of Genetics and Genomic Medicine, UCL Great Ormond Street Institute of Child Health, University College London, London, UK; 3 Camden and Islington NHS Foundation Trust, London, UK; 4 Epidemiology and Applied Clinical Research Department, Division of Psychiatry, University College London, London, UK; 5 Servicio de Psiquiatría, Hospital Provincial de Zamora, Instituto de Investigación Biomédica de Salamanca, Zamora, Spain; 6 Department of Intensive Care (4131), Copenhagen University Hospital – Rigshospitalet, Copenhagen, Denmark; 7 Clinical Pharmacological Unit, Zealand University Hospital, Roskilde, Denmark; 8 Institute of Clinical Medicine, Copenhagen University, Copenhagen, Denmark; 9 Research Unit for Clinical Psychopharmacology, Psychiatry West, Slagelse, Denmark; 10 Department of Health Services and Population Research, Institute of Psychiatry, Psychology and Neuroscience, King’s College London, London, UK; 11 King’s Health Economics, Institute of Psychiatry, Psychology and Neuroscience, King’s College London, London, UK

**Keywords:** Pharmacogenetics, Genetics research

## Abstract

Pharmacogenomics could optimize antipsychotic treatment by preventing adverse drug reactions, improving treatment efficacy or relieving the cost burden on the healthcare system. Here we conducted a systematic review to investigate whether pharmacogenetic testing in individuals undergoing antipsychotic treatment influences clinical or economic outcomes. On 12 January 2024, we searched MEDLINE, EMBASE, PsycINFO and Cochrane Centrale Register of Controlled Trials. The results were summarized using a narrative approach and summary tables. In total, 13 studies were eligible for inclusion in the systematic review. The current evidence base is either in favor of pharmacogenetics-guided prescribing or showed no difference between pharmacogenetics and treatment as usual for clinical and economic outcomes. In the future, we require randomized controlled trials with sufficient sample sizes that provide recommendations for patients who take antipsychotics based on a broad, multigene panel, with consistent and comparable clinical outcomes.

## Abstract (web summary)

The authors conducted a systematic review to examine whether pharmacogenetic testing affects clinical or economic outcomes in patients taking antipsychotic medication.

## Main

Psychotic disorders affect about 3% of the population^1^ and entail a major economic burden for health services. A recent systematic review indicated that the annual societal cost of schizophrenia varies per patient, from US$819 in Nigeria to US$94,587 in Norway^2^.

Antipsychotic drugs have demonstrated efficacy, and like almost every medication, they are prescribed in a prioritized order based on our knowledge of their tolerability and are adapted to the patient’s needs using clinical observations to identify the optimal medication and dose that will maximize response and minimize toxicity^3^. However, this process can lead to substantial delays in finding the drug and dose of choice for each patient^3^ because the response to antipsychotics is highly variable among individuals^4^. While the majority of patients diagnosed with schizophrenia experience symptom improvements with antipsychotics, approximately 34% of patients are ‘treatment-resistant’, indicating a limited or lack of response to at least two trials of an antipsychotic therapy at an appropriate dose^4,5^. In addition, antipsychotic drugs have a plethora of adverse drug reactions (ADRs), some of which are serious and thought to contribute to the excess mortality associated with severe mental illness^6^.

The interindividual variability in response to antipsychotic therapy is partly explained by genetics in conjunction with clinical, demographic and environmental factors^7^. Indeed, cytochrome P450 (CYP450) are a superfamily of enzymes that are involved in the metabolism of drugs^3^, and the genes coding CYP isoforms are highly polymorphic^8^. Based on an individual’s genotype, studies and guidelines classify individuals into metabolic phenotypes: poor metabolizers, intermediate metabolizers, normal metabolizers, and rapid or ultrarapid metabolizers, which correspond to individuals carrying deleted or defective, partially defective, normal, duplicate or higher expression of CYP genes, respectively. These genetic variants may impact enzyme activity, which could affect the rate of clearance of antipsychotics, and possibly an individual’s response and adverse reactions^3,9^.

Knowledge of patients’ drug metabolic status through pharmacogenetic testing might optimize the selection of medication and adjustment of doses^9^. A systematic review of qualitative and quantitative studies by Hansen et al.^10^ underlined the potential benefits of pharmacogenetic testing for any medication, from patients’ and clinicians’ perspectives. Patients felt that pharmacogenetics would increase their confidence with the choice of drug, therefore motivating them to adhere to their medication plan. Ongoing adherence is key to optimal outcomes in patients, but up to 75% of patients at 2 years post hospital discharge are nonadherent with antipsychotic medication^11^. Nonadherence is associated with worse prognosis, increased frequency of relapse, rehospitalization and, therefore, increased utilization of healthcare resources and costs^12^. In addition, Swen et al.^13^ reported that the pharmacogenetics-guided treatment with an index drug (that is, any drug with recommendations in the guidelines of the Dutch Pharmacogenetics Working Group, including antipsychotics as well as other drugs, such as antidepressants, anticoagulants and analgesics, among others) using a 12-gene panel significantly reduced the incidence of developing an ADR by 30%. Other pharmacogenetic studies covering a wide range of drugs have been conducted^14^, and many have similarly reported improved tolerability^15^, reduced symptom severity^16,17^ and reduced healthcare costs^18,19^.

Fleeman et al.^20^ conducted a systematic review for pharmacogenetic testing in adults taking antipsychotics over a decade ago. They confirmed the compelling biological evidence supporting CYP450 genetic testing as well as analytical validity and accuracy of assays but did not identify any observational or randomized studies that investigated its clinical utility or cost-effectiveness. In this Analysis, considering recent technological and research advancements, we conducted a systematic review to investigate whether pharmacogenetic testing for individuals undergoing antipsychotic treatment influences clinical or health economic outcomes.

## Results

### Inclusion and exclusion of studies

**Fig. 1:**
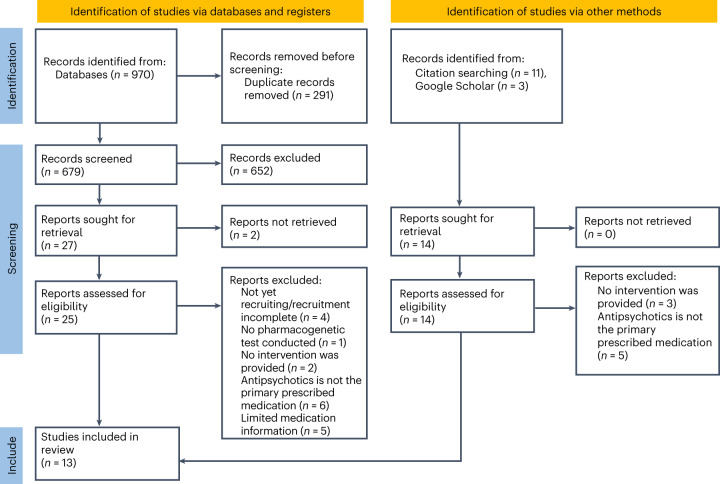
**PRISMA flow diagram**.

The database search yielded 970 publications: EMBASE (*n* = 530), MEDLINE (*n* = 242), PsycInfo (*n* = 100) and Cochrane Library (*n* = 98) (Fig. 1). After removing duplicates and screening on the basis of titles and abstracts, we were left with 25 potentially eligible studies. After applying the prespecified inclusion criteria to the full-text articles, seven studies remained. An additional 14 potentially eligible studies were identified from manual screening of citations and Google Scholar. After assessing for eligibility, six studies remained. Information about the excluded studies is detailed in Supplementary Table 1. In total, 13 eligible studies were included in the systematic review (Fig. 1). Table 1 summarizes the design and key findings from each of the studies included.

**Table 1:** Summary of study characteristics

Authors	Year	Country	Study design	Sample characteristics	Outcomes measured	Medication	Genes
Studies with three comparators							
Jürgens et al.^26^	2020	Denmark	Single-blind RCT Three arms: pharmacogenetics versus SCM versus TAU Assessment points: baseline and 12 months	Total *n* = 161. Pharmacogenetics (*n* = 84) versus TAU (*n* = 77) Age, median (years): 42 versus 42 Sex (% female): 43 versus 46 Ethnicity: N/A Medication: N/A Diagnosis (%): paranoid schizophrenia, 72 versus 65; schizotypal disorder, 20 versus 20; persistent delusional disorders, 3 versus 2; acute and transient psychotic disorders, 1 versus 2; schizoaffective disorders, 2 versus 8	Antipsychotic drug persistence, that is, days to first modification of the initial treatment Number of drug and dose changes Adverse drug response (UKU) Symptom severity (using SAPS) Compliance (ROMI)	Antipsychotics	*CYP2D6* and *CYPC19*
Studies with two comparators							
Kang et al.^24^	2023	China	Double-blind RCT Two arms: pharmacogenetics versus TAU Assessment points: baseline and 6 weeks	Total *n* = 210 Pharmacogenetics (*n* = 113) versus TAU (*n* = 97) Age, median (years): 29.9 versus 28.3 Sex (%): male, 100 Ethnicity (%): Han Chinese, 100 Medication: quetiapine, 26.5 versus 19.6; risperidone, 31.0 versus 45.4; olanzapine, 13.3 versus 11.3; aripiprazole, 15.0 versus 8.2; ziprasidone, 0.9 versus 3.1; paliperidone, 6.2 versus 7.2; clozapine, 1.8 versus 2.1; amisulpride, 5.3 versus 3.1. Diagnosis (%): schizophrenia, 100	Symptom severity (using PANSS) ADRs	Antipsychotics	*CYP1A2*, *CYP2D6*, *CYP3A4*, *DRD2*, *EPM2A*, *HTR1A*, *HTR2A*, *HTR2C*, *MC4R*, *RGS4* and *SH2B1*
Herbild et al.^28^	2013	Denmark	Double-blind RCT Three arms: pharmacogenetics versus extensive clinical monitoring versus TAU Time horizon: 1 year	Total *n* = 207 Pharmacogenetics (*n* = 103) versus TAU (*n* = 104) Age, mean (years): 41 versus 42 Sex (% female): 45 versus 44 Ethnicity: N/A Medication: N/A Diagnosis (%): schizophrenia, 74 versus 71; schizotypal disorders, 24 versus 21; other disorders, 5 versus 12	Pharmaceutical costs Hospitalization costs	Antipsychotics	*CYP2D6* and *CYP2C19*
Arranz et al.^27^	2019	Spain	Double-blind RCT Two arms: pharmacogenetics versus TAU Assessment points: baseline and 12 weeks	Total *n* = 290. Pharmacogenetics (*n* = 123) versus TAU (*n* = 167) Age, median (years): 46.1 versus 48.7 Sex (% female): 48.8 versus 43.7 Ethnicity: N/A Medication (%): clozapine, 35 versus 52.7; risperidone, 13 versus 12; olanzapine, 20.3 versus 8.4; paliperidone, 13 versus 13; aripiprazole, 5.7 versus 7.8; quetiapine 8.9 versus 3; ziprasidone, 0.8 versus 1.2; trifluoperazine, 0.8 versus 0.6; haloperidol, 0.8 versus 0.6; asenapine, 0.8 versus 0.6; pimozide, 0.8 versus 0. Diagnosis (%): schizophrenia, 86 versus 69; schizoaffective, 5 versus 4; delusional disorder, 9 versus 27	Symptom severity (using PANSS) ADRs (using UKU)	Antipsychotics	*CYP2D6*, *CYPC19*, *CYP1A2* and *CYP3A5*
Arranz et al.^21^	2022	Spain	Prospective observational study Two arms: pharmacogenetics versus TAU Assessment points: baseline and 4 months	Total *n* = 104. Pharmacogenetics (treatment resistant) (*n* = 42) versus TAU (*n* = 62) Age, mean (years): 18.79 versus 13.83 Sex (% female): 26% versus 8% Ethnicity: N/A Medication (%): antipsychotics, 67 versus 32; antidepressants, 48 versus 11; anxiolytics, anticonvulsants and others, 26 versus 56; no current medication, 7 versus 0 Diagnosis: 100% autism spectrum disorder	Symptom severity (using CGI and CGAS)	Antipsychotics, antidepressants, anxiolytics and anticonvulsants	*CYP1A2*, *CYP2C19*, *CYP2D6* and *SLC6A4*
Studies with one comparator							
Carrascal-Laso et al.^22^	2020	Spain	Retrospective observational study One arm: pharmacogenetics only Time horizon: 3 years	Total sample (*n* = 188) offered pharmacogenetics test. Age, median (years): 47 Sex (% female): 37.8% Ethnicity: N/A Medication (%): N/A Diagnosis (%): dementia, 0.53; substance-related disorder 6.38; schizophrenia, 67.02; persistent delusional disorder, 1.06; brief and acute psychotic disorder, 0.53; schizoaffective disorder, 6.92; bipolar disorder, 13.30; major depressive disorder, 0.53; specific personality disorder, 1.06; mixed personality disorder, 0.53; intellectual disability, 1.06	Mean daily dose Polytherapy cases	Antipsychotics	*CYP1A2*, *CYP2B6*, *CYP2C9*, *CYP2C19*, *CYP2D6*, *CYP3A5* and *ABCB1*
Carrascal-Laso et al.^23^	2021	Spain	Retrospective observational study One arm: pharmacogenetics only Time horizon: 3 years	Total sample (*n* = 188) offered pharmacogenetics test. Age, median (years): 47 Sex (% female): 37.8% Ethnicity: N/A Medication (%): N/A Diagnosis (%): dementia, 0.53; substance-related disorder 6.38; schizophrenia, 67.02; persistent delusional disorder, 1.06; brief and acute psychotic disorder, 0.53; schizoaffective disorder, 6.92; bipolar disorder, 13.30; major depressive disorder, 0.53; specific personality disorder, 1.06; mixed personality disorder, 0.53; intellectual disability, 1.06	Pharmaceutical costs Hospitalization costs	Antipsychotics	*CYP1A2*, *CYP2B6*, *CYP2C9*, *CYP2C19*, *CYP2D6*, *CYP3A5* and *ABCB1*
Walden et al.^25^	2019	Canada	Prospective observational study One arm: pharmacogenetics only Assessment points: baseline, 6 weeks and 12 weeks	Total sample (*n* = 80) offered pharmacogenetics test. Age, mean (years), 43 Sex (% female): 43.8% Ethnicity/race (% of participants): European Caucasian 68.8%, African 3.8%, Asian 3.8%, others 12.5%, mixed 11.3% Medication (%): antipsychotics, 47.5; antidepressants, 23.8; anxiolytics, 7.5; antipsychotics and antidepressants, 11.3; antipsychotics, antidepressants, and anxiolytics, 6.3; no medication, 3.8 Diagnosis: schizophrenia/schizoaffective, 53.8%; anxiety/depression, 40%, others, 6.3%	Physician’s opinions (using PIP-FQ) ADRs (UKU)	Antidepressants, anxiolytics and antipsychotics	*CYP2D6* and *CYP2C19*
Markov/decision models							
Ninomiya et al.^31^	2022	United Kingdom	Decision tree with Markov model Third-party payer perspective Two arms: pharmacogenetics versus TAU Time horizon: 10 years	The target population was adult men and women with treatment-resistant schizophrenia	ICER	Clozapine	*SLCO1B3*–*SCLO1B7*, *HLA-DQB1* and *HLA-B*
Girardin et al.^35^	2019	United States	Decision tree with semi-Markovian model Third-party payer perspective Three arms: (1) PGx-guided clozapine treatment with ANCM for patients who test positive for one or both alleles, (2) PGx-guided clozapine treatment for patients who test negative or alternative antipsychotics for patients who test positive, (3) TAU. Time horizon: 3 years	The target population was adult men and women with treatment-resistant schizophrenia	ICER	Clozapine	*HLA-DQB1* and *HLA-B*
Kurylev et al.^29^,	2018	Russia	Decision tree Three arms: (1) PGx in 100% of patients, (2) PGx in 30% of patients, (3) TAU. Time horizon: N/A	The target population was patients diagnosed with paranoid schizophrenia	Hospitalization costs Medication costs	Antipsychotics	*CYP2D6*
Rejon-Parrilla et al.^32^,	2014	United Kingdom	Decision tree with Markov model Healthcare provider perspective (NHS) Two arms: (1) traditional dosing, (2) pharmacogenetic testing Time horizon: 2 years	The target population was previously untreated patients newly diagnosed with schizophrenia, aged 25	Incremental cost-effectiveness	Risperidone	*CYP2D6*
Perlis et al.^34^,	2005	United States	Decision tree with Markov model Societal perspective Three arms: (1) no PGx test, clozapine as first-line treatment, (2) PGx testing, clozapine as first line if they test positive for or third line if the test negative, (3) no PGx testing, clozapine as third line. Time horizon: lifetime	The target population was a 30-year-old patient with schizophrenia	Incremental cost-effectiveness	Clozapine	N/A

N/A indicates that information was not reported in the original article. ANCM, absolute neutrophil count monitoring; CGAS, Children’s Global Assessment Scale; PGx, pharmacogenetics; SCM, structural clinical monitoring.

### Study characteristics

The sample size of the studies ranged from 80 to 290 participants, and the average age ranged from 14 to 49 years. Regarding gender, most studies were well balanced, except three studies that included less than 40% female participants^21–23^ and one study that included only male participants^24^. Most studies were conducted in Europe and North America, although there was one study conducted in China^24^. Only two studies reported the ethnicity or ancestry of their participants^24,25^. The primary diagnosis among the studies was a psychotic disorder (schizophrenia, schizotypal disorder, schizoaffective disorder, persistent delusional disorder, brief and acute psychotic disorder, and bipolar disorder). However, one study focused on patients with a diagnosis of autism spectrum disorder^21^, and one study included patients with different diagnoses, including schizophrenia, anxiety and depression (although schizophrenia accounted for over 50% of the diagnoses in this sample)^25^. Including the decision/Markov models, four studies had three comparators (for example, pharmacogenetics versus extensive clinical monitoring versus treatment as usual (TAU)), six studies had two comparators (for example, pharmacogenetics versus TAU) and three studies had one group (pharmacogenetics only). Several studies focused exclusively on antipsychotics (*n* = 11), while others focused on antipsychotics as well as other psychotropic medications, as part of a broader combinatorial treatment (*n* = 2). The genes included in the pharmacogenetic tests varied widely, but the *CYP2D6* gene was included in many studies. There were no industry-funded studies included in the review.

### Clinical outcomes

**Fig. 2:**
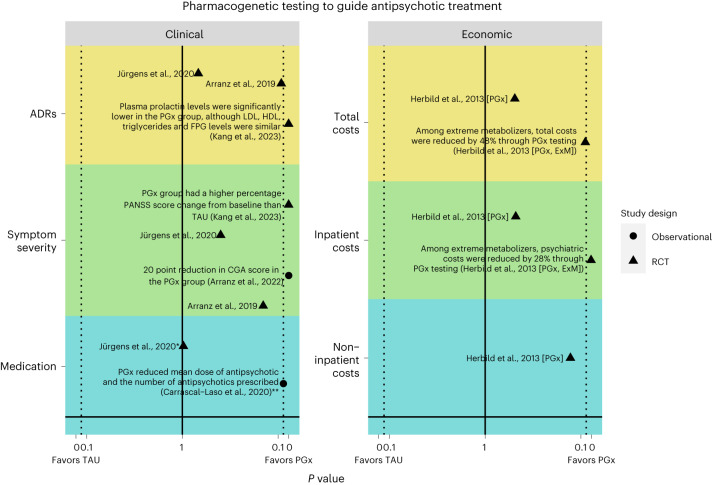
Visualization of the literature with key results for the clinical and economic outcomes. Primary studies that reported a *P* value are plotted to depict the direction of effect for each outcome (whether they favor pharmacogenetics or TAU or whether there is no significant difference between the two treatment arms). The *y* axis lists the outcomes grouped by themes. The *x* axis plots the *P* value reported in the primary study as a measure of the strength of the evidence. The solid line marks a *P* value of 1, and the dotted line marks the significance threshold of *P* < 0.05. The study design (RCT or observational) and sample size are displayed. Herbild et al.^28^conducted a main analysis comparing PGx versus TAU (denoted [PGx]) and a subanalysis comparing extreme metabolizers in the PGx group (denoted [PGx, ExM]) to TAU. For non-inpatient costs (primary care costs) there was no subgroup analysis for the extreme metabolizers. Studies that did not report *P* values were excluded from the visualization. FPG, fasting plasma glucose; PGx, pharmacogenetics; RCT, randomized control trial. *Exact *P* value not indicated but specified that it is >0.05; **exact *P* value not indicated but specified that it is <0.05.

Overall, there were four randomized controlled trials (RCTs), two retrospective studies and two prospective studies that reported clinical outcomes. Studies reported ADRs, symptom severity, medication, hospitalizations, polypharmacy and physicians’ opinions (Table 2). The results for the different clinical outcomes are visualized in Fig. 2.

**Table 2:** Clinical outcomes included in the systematic review and their corresponding definition/measure of the outcome

Outcome	Definition/measure of outcome
ADRs	UKU adverse effects score^26,27^
Symptom severity	SAPS^26^ PANSS^27^ CGI-S^17,21^ Children’s Global Assessment Scale (CGAS)^21^
Clinicians’ opinions	PIP-FQ^25^
Hospitalizations	Overall hospitalization stays per patient^23^
Medication prescribing	Antipsychotic drug persistence, measured as time in days to the first modification of the initial antipsychotic treatment (drug or dose change), to indicate tolerability of medication^26^ Drug changes^26^ Dose changes by visual inspection of temporal dose-adjustment graphs^26^ Mean daily dose^22^ Polytherapy through the number of antipsychotics prescribed^22^

### ADRs

Two studies assessed ADRs using the Udvalg for Kliniske Undersøgelser (UKU) side effect rating scale, neither of which found a statistically significant difference in UKU score between the two treatment arms (pharmacogenetics versus TAU)^26,27^. Kang et al.^24^ did not identify a significant difference in metabolic profiles (triglycerides, low-density lipoprotein (LDL) and high-density lipoprotein (HDL)cholesterol, and fasting plasma glucose) between the intervention and TAU group, except plasma prolactin levels, which were significantly lower in the intervention group compared to the control at the end of week 12 (29.4 ng ml^−1^ in the pharmacogenetics group versus 40.4 ng ml^−1^ in TAU, *P* = 0.03).

### Symptom severity

Symptom severity was reported using the Scale for the Assessment of Positive Symptoms (SAPS), Positive and Negative Symptoms Scale (PANSS), Clinical Global Impression-Severity (CGI-S) and Children’s Global Assessment (CGA) scale. Jürgens et al.^26^ and Arranz et al.^27^ did not identify a significant difference in the change in symptom severity in the pharmacogenetics group compared to TAU. In contrast, Kang et al.^24^ found that the pharmacogenetics group had a higher percentage PANSS score change from baseline than the TAU group at the end of week 6 (74.2% versus 64.9%; 95% confidence interval (CI) 4.4 to 14.1 percentage points; *P* < 0.001). In their study, the response rate at the end of week 6 was significantly higher in the pharmacogenetic group (82.3%) compared to TAU (64.9%) (adjusted odds ratio (OR) 2.48; 95% CI 1.28 to 4.80, *P* = 0.01). Similarly, the rates of symptomatic remission at the end of week 12 were also significantly higher in the pharmacogenetics group (62.8%) compared to TAU (45.4%) (adjusted OR 2.03; 95% CI 1.11 to 3.60, *P* = 0.02). Arranz et al.^21^ also identified an improvement in symptomology: 39 treatment-resistant patients (93%) demonstrated improvement in their CGI scores, and 37 (88%) showed improvements in their CGA scores. Indeed, after pharmacogenetic testing, a 2- and 20-point average improvement in CGI and CGA scores was identified for the pharmacogenetics group, respectively (*P* = 1 × 10^−5^ for CGI scores, *P* = 5 × 10^−8^ for CGA scores).

### Clinicians’ opinions

Physicians’ opinions were evaluated using the Pharmacogenetics in Psychiatry Follow-up Questionnaire (PIP-FQ) by Walden et al.^25^. The PIP-FQ revealed that 23% (*n* = 14) of physicians concluded that their patients improved after pharmacogenetics testing for *CYP2D6* and *CYP2C19* genes. The remaining physicians concluded that the patients did not change (*n* = 25), their patients were not assessed (that is, due to a lack of follow-up appointment with the patient) (*n* = 21) or no answer was provided (*n* = 20).

### Hospitalization

Carrascal-Laso et al.^23^ demonstrated that, before applying the pharmacogenetics test, participants in the study accounted for 504 hospitalization stays. This was reduced to 218 hospitalizations after adjusting treatment on the basis of the pharmacogenetics test. Arranz et al.^21^ also found that pharmacogenetic testing led to a reduction in the visits to their clinicians (ten fewer visits per patient per year) and a reduction in hospital stays (total reduction of 3 months in hospital stays).

### Medication prescribing

Jürgens et al.^26^ found no difference in antipsychotic drug persistence (number of days until a medication or dose change) in the pharmacogenetics group compared to TAU, even in a subgroup analysis including only extreme metabolizers (poor and ultrarapid metabolizers for *CYP2D6* and/or *CYP2C19* genes). However, Jürgens et al.^26^ showed that extreme metabolizers in the intervention group experienced fewer drug and dose changes than the TAU group (pharmacogenetic group, *β* = −1.2; 95% CI −4.1 to 1.2; TAU, *β* = −2.3; 95% CI −5.0 to 0.4). Carrascal-Laso et al.^22^ demonstrated that the average number of antipsychotics prescribed per patient reduced from 1.82 at baseline to 1.27 after pharmacogenetics testing, and this change was statistically significant (*P* < 0.05). Similarly, at baseline, almost 21% of patients were prescribed more than five drugs (any mental/physical health drugs), which was reduced to less than 11% post-pharmacogenetics testing, again a significant reduction in polypharmacy (*P* < 0.05).

### Economic outcomes

Overall, there were two study-based economic evaluations (using patient-level data) and five model-based economic evaluations (using data from existing literature). Most of these were cost-effectiveness analyses (*n* = 4), as well as a few cost analyses (*n* = 2). There was also one study that conducted a cost–benefit analysis. Among these studies, two studies were conducted from a third-party perspective, one from a healthcare payer system perspective and one from a society perspective. The remaining studies did not specify the perspective (*n* = 3). Moreover, the time horizon employed varied widely, including 1 year (*n* = 1), 2 years (*n* = 1), 3 years (*n* = 2) and 10 years (*n* = 1). There was one study that did not specify a time horizon. Economic outcomes included overall cost of healthcare resource utilization, inpatient costs (hospitalizations), non-inpatient costs (primary care and pharmaceutical costs) and incremental cost-effectiveness ratio (ICER). The results for the economic outcomes are visualized in Fig. 2.

### Overall healthcare costs

Herbild et al.^28^ demonstrated that there was no statistically significant difference in total costs between the pharmacogenetics and TAU group. However, total costs were 177% higher in the extreme metabolizers (poor and ultrarapid metabolizers for *CYP2D6* and/or *CYP2C19* genes) than among the normal metabolizers; this difference was reduced by 48% among extreme metabolizers in the intervention group (*P* = 0.058). Moreover, Carrascal-Laso et al.^23^ found that pharmacogenetics testing was associated with a reduction in total costs for 67% of the patients.

### Inpatient costs

Regarding inpatient costs, such as the costs attributed to services in the psychiatric hospital sector, Herbild et al.^28^ showed that there was no difference between the pharmacogenetics and TAU group. However, extreme metabolizers were incurring significantly higher costs than normal metabolizers; these excess costs in the extreme metabolizers were significantly reduced by 28% through pharmacogenetic testing (*P* < 0.05). Furthermore, no difference was identified for the nonpsychiatric hospital costs between the intervention and TAU group. Carrascal-Laso et al.^23^ found that total hospital costs decreased from US$2,335 before pharmacogenetics testing (2013–2015) to US$948 after pharmacogenetics testing (2016–2019), which is a 59% reduction. This was supported by a pharmacoeconomic model by Kurylev et al.^29^ that found that pharmacogenetic testing reduced the length of stay of patients in hospital, which translated to a total reduction in hospital costs by 382,433 Russian Rubles.

### Non-inpatient costs

Carrascal-Laso et al.^23^ found that the pharmacogenetics intervention led to a reduction of 10% (before versus after pharmacogenetics, US$3,142 versus US$2,827 per patient per year) in pharmaceutical costs. No statistically significant cost difference was identified by Herbild et al.^28^ between the intervention and TAU group for primary care services; there was no subgroup analysis for the extreme metabolizers.

### ICER

The ICER is the difference in mean costs of two interventions (that is, a new intervention and the standard intervention) divided by the difference in mean health effects, such as quality-adjusted life years (QALY)^30^. Ninomiya et al.^31^ compared pharmacogenetics-guided clozapine treatment to TAU and calculated an ICER of £16,215 per QALY, that is, it would cost an extra £16,215 to gain an additional QALY if the patient were prescribed antipsychotics using the pharmacogenetics-guided strategy as opposed to the traditional strategy. Similarly, Rejon-Parrilla et al.^32^ found that pharmacogenetic testing entailed an additional cost of £19,252 per QALY. Both of these values remain below the conventional decision threshold of £20,000 per additional QALY gained outlined by the National Institute for Health and Clinical Excellence^30,33^. Perlis et al.^34^ compared pharmacogenetics-guided clozapine treatment as first-line treatment for individuals who test negative for genetic variants in neurotransmitter-receptor-related genes (*5-HT*_*2A*_, *5-HT*_*2C*_, *5-HTTLPR* and *H2*), to TAU, involving no testing and clozapine as a third-line treatment. They identified a reduced likelihood of treatment failure and relapse for the pharmacogenetics-guided group taking clozapine as a first-line treatment. Overall, they found that pharmacogenetic testing yields a cost of US$47,705 per QALY gained, compared to TAU, which is below the conventional decision threshold of US$50,000 per additional QALY gained. Finally, Girardin et al.^35^ compared TAU to pharmacogenetics-guided clozapine treatment that would involve absolute neutrophil count monitoring only for patients who test positive for one or both susceptibility alleles. They reported an ICER of $3.9 million per QALY, meaning that TAU cost an extra US$3.93 million (95% CI 2.01 to 8.17) per additional QALY gained compared to the pharmacogenetic strategy. The results of these studies were primarily sensitive to the pharmacogenetic test parameters, such as sensitivity and cost, as well as clozapine-induced agranulocytosis prevalence and infection-related death rates.

### Quality assessment

Quality assessment was conducted using the Downs and Black checklist for RCTs and non-RCTs that reported a clinical outcome, and results varied from 15 to 24 (out of 27), with a mean score of 19.7 (Supplementary Table 2). The studies demonstrated a good ability to report the study objectives, methods, sample characteristics and main findings. However, details regarding patients lost to follow-up was poorly described in 50% of the studies (*n* = 3). More than half of the participants in the studies were not blinded to the intervention (*n* = 4), and there was no attempt to blind those measuring the main outcomes in 50% of studies (*n* = 3). Moreover, in at least half of the studies, participants were not randomized to intervention groups (*n* = 3), randomization was not concealed from both patients and staff until recruitment was complete (*n* = 4) and there was inadequate adjustment for confounding (*n* = 3).

Quality assessment was also conducted for economic evaluations using the Consolidated Health Economic Evaluation Reporting Standards (CHEERS) checklist, and results varied widely. Total scores ranged from 43% to 75%, with a mean score of 62% (Supplementary Table 3). For most of the studies, a clear title, abstract and background was provided, findings were summarized effectively in the results, and a comprehensive discussion was provided. However, reporting of methodology was weaker: none of the studies provided a health economic analysis plan; three studies did not clearly outline their methods for analysis; four studies failed to report or justify their chosen time horizon; five studies did not report or justify their chosen discount rate or perspective; heterogeneity was characterized by only one study; and none of the studies incorporated patient and public involvement in the design of the study. Furthermore, sources of funding could have been more transparent as several studies did not specify funding (*n* = 3). We assessed certainty of the evidence using the Grading of Recommendations, Assessment, Development, and Evaluation (GRADE) guidelines, which demonstrated low certainty for most outcomes (Supplementary Table 4).

## Discussion

In this systematic review, we highlighted several important findings. Firstly, clinical outcomes showed either no difference with TAU or a benefit in favor of pharmacogenetics, although there was stronger evidence of clinical utility when pharmacogenetic testing was conducted using a multigene panel. We hypothesize that pharmacogenetic testing for antipsychotics using a multigene panel, such as the 11-gene panels used by Kang et al.^24^, increases the frequency of actionable variants in the sample, which increases statistical power to detect differences between the intervention and TAU groups. Similarly, pharmacogenetics testing either demonstrated no difference in costs or a reduction in overall, inpatient and outpatient costs, compared to TAU, particularly for extreme metabolizers that were suggested to incur higher costs.

Quality assessment of RCTs and non-RCTs using the Downs and Black checklist revealed several methodological limitations. Firstly, several studies were not blinded and/or randomized. There was an underestimation of the confounding factors, as studies did not consider that participants who opt to undergo pharmacogenetic testing may be more engaged (selection bias) and, therefore, have greater adherence, or that the effect of closer monitoring by the clinicians may increase patients’ adherence; this confounder was addressed only by Jürgens et al.^26^, who included three arms in their study: pharmacogenetics-guided group, TAU and structured clinical monitoring, in which the patients’ primary contact person systematically recorded adverse effects and factors affecting the patient’s adherence at least once quarterly. Finally, the studies were limited by statistical power due to small sample sizes, as all the studies had less than 300 participants. The CHEERS checklist for economic evaluations revealed that several studies failed to report or justify their chosen perspective, time horizon and discount rates. There was also no consideration of how findings may vary for subgroups, except by Herbild et al.^28^, who explored healthcare costs for extreme metabolizers. Thus, based on the quality assessment of the included studies, the results should be interpreted with caution.

The widespread implementation of pharmacogenetics has yet to occur in most healthcare systems globally and has predominantly been restricted to academic and other highly specialized centers^36^. Nonetheless, an important milestone for pharmacogenetics in the United Kingdom has been the implementation of routine screening for four dihydropyrimidine dehydrogenase variants associated with toxicity for fluoropyrimidine chemotherapy into the National Health Service in 2020 to reduce the development of ADRs^37^.

Similarly, antipsychotic medications are associated with increased incidence of ADRs, such as clozapine, which makes it a drug requiring mandatory full-blood count monitoring due to the risk of neutropenia and agranulocytosis^38^. A meta-analysis demonstrated that individuals carrying the HLA-DRB1*04:02 allele had nearly sixfold-higher odds of clozapine-induced agranulocytosis^39^. In addition, a recent retrospective study found that, in a cohort of patients taking clozapine, 4.3% reported minor neutropenia and 1.2% reported serious neutropenia leading to cessation of clozapine^40^. While clozapine reduces the mortality rate in severe schizophrenia by reducing the suicide rate, it may increase the mortality rate for common causes of death, such as pulmonary embolism and cardiac problems^41^. Thus, pharmacogenetics could perhaps benefit this patient population to reduce the incidence of adverse events in patients who take clozapine, and this requires further investigation.

There is a considerable need to invest in mental health research, specifically in research that improves service users’ care and quality of life^42,43^. This systematic review has revealed a limited number of studies with sufficient sample sizes that contain clinical and/or economic data; thus, further research is warranted to address the specific benefits of pharmacogenetic testing for patients. In addition, a recent report by the Royal College of Psychiatrists indicated that pharmacogenetic testing cannot be recommended for psychotropic medication due to gaps in the literature, such as insufficient evidence of clinical utility^44^. Despite the need for further research in this field, mental health research globally receives less funding than research into physical conditions. Indeed, the median government spending on mental health around the world per capita in 2017 was US$2.50 (ref. ^42^). Furthermore, mental health research funding is predominantly allocated to biological and etiological research, which makes up over 50% of funding, and only 7% to health services, clinical and prevention research, each^45^.

To our knowledge, this systematic review is the first to evaluate whether pharmacogenetic testing for antipsychotic medication may improve clinical and/or economic outcomes and to assess the quality and certainty of the findings. In addition, the authors are not affiliated with industry, which reduces bias. However, our study had several limitations. First, the scope of this review was wide due to the scarcity of the data. This meant that there was heterogeneity among the studies due to differences in study design (RCTs and non-RCTs with multiple different comparators) and outcomes measured, particularly for clinical outcomes that were assessed using many different clinical scales. Second, the search picked up very few studies from outside of Europe and North America, indicating limited clinical generalizability of the findings, therefore highlighting an important gap in the literature that should be addressed in future research. This is important because the prevalence of schizophrenia is high in East and South Asia, with a patient population of approximately 7.2 and 4.0 million^46^. In addition, compared to Caucasian cohorts, these populations have different frequencies of variants for CYP450 enzymes. For example, while CYP2D6*10 is the most abundant allele in East Asian populations (minor allele frequency 58.7%), this allele is considerably less common in Europeans (minor allele frequency 0.2%)^47^. Thirdly, not all antipsychotics have pharmacogenetic recommendations, which would further reduce the ability to detect differences.

Overall, the current evidence base shows either no difference or is in favor of pharmacogenetics-guided prescribing for clinical and economic outcomes. To support the clinical implementation of pharmacogenetics testing into routine mental healthcare, RCTs with sufficient sample sizes that provide recommendations for patients who take antipsychotics based on a broad, multigene panel are required, with consistent and comparable clinical outcomes.

## Methods

The systematic review was registered with PROSPERO (registration ID: CRD42023380454) and was performed according to the Preferred Reporting Items for Systematic Reviews and Meta-Analyses (PRISMA) 2020 guidelines^48^. This study involved the use of data from other studies, and therefore did not require ethics approval as ethics approval was obtained in the original studies.

### Eligibility criteria

On 12 January 2024, we searched for studies that evaluated clinical and/or economic outcomes after pharmacogenetics-guided treatment in a sample of individuals taking antipsychotics. No limits were applied on patients’ age or diagnosis. No restrictions by country, healthcare setting or monetary currency were applied. No restrictions were imposed on date range or language, but the search was conducted in English. Studies were excluded if antipsychotics were not the primary prescribed medication and if they were a protocol, review, commentary, letter or editorial.

### Search strategy

Several electronic databases were searched to identify relevant articles: MEDLINE (via Ovid), EMBASE (via Ovid), PsycINFO (via Ovid) and Cochrane Centrale Register of Controlled Trials. The following search string was used: (antipsychotic*) AND (pharmacogenetic* OR pharmacogenomic* OR pharmacogenetics OR genetic test*) AND ((prospective OR randomi* OR trial OR intervention) OR (cost and (effect* or benefit* or utility or utilities or outcome* or analysis or analyses or consequence* or minimi*))). Furthermore, a manual search of the reference lists of the included articles and relevant existing reviews and a manual search of papers that have referenced the included articles using Google Scholar Citations was conducted.

### Study selection

The first stage of the study selection involved collating articles that appeared eligible from the title and abstract or were of unclear eligibility. The titles and abstracts were initially assessed by independent reviewers N.S.K., S.R., G.M. and G.H. using Rayyan^49^. The second stage involved screening full-text articles to determine if the studies met the eligibility criteria. Any discrepancies were resolved by consulting an additional independent reviewer, E.B.

### Data extraction and presentation of results

The data were extracted from the selected studies using a custom data extraction template in Excel. The extracted data included the following: study authors, year of publication, study title, study design, country, sample size, sample characteristics, test gene composition and outcomes measured. A narrative approach was adopted due to the substantial heterogeneity between the included studies.

Certainty of the evidence was rated by N.S.K. using the GRADE guidelines^50^, which assessed the following domains for each outcome: risk of bias, inconsistency, indirectness, imprecision and publication bias. A total score was determined to measure certainty: high (≥4 points, high certainty that the true effect is close to the estimated effect), moderate (3 points), low (2 points) or very low (≤1, the true effect is probably different from the estimated effect).

### Quality assessment

Quality assessment was conducted by N.S.K. using a modified Downs and Black checklist for RCTs and non-RCTs that assessed clinical outcomes^51^. The modified checklist includes 26 items that assess various methodological components, such as reporting, external validity, internal validity and power. Each item was either awarded one point if the criteria were met or no points if the criteria were not met, except item 5. This item assessed whether the principal confounders in each group of subjects were clearly described and was awarded one point if the criteria were partially met or two if the criteria were fully met. If the item could not be inferred from the study, it was marked as ‘unable to determine’. In total, studies are awarded a total score ranging from 0 to 27.

Moreover, the quality of economic evaluations were assessed separately using the CHEERS 2022 checklist^52^. The checklist consists of 28 items, and each item is awarded a point if the criteria were met, or no points if the criteria were not met or only partially met. If the item was not applicable to the study (for example, a cost-minimization analysis could not be assessed by items 11–13, which assess the selection, measurement and valuation of health outcomes), the item was marked ‘N/A’. The total percentage score was calculated.

### Reporting summary

Further information on research design is available in the Nature Portfolio Reporting Summary linked to this article.

### Supplementary information

Supplementary Information Supplementary Tables 1–4.

Reporting Summary

## Data Availability

Data used (means, effect sizes, standard deviations and confidence intervals) can be obtained from the original studies in the systematic review, listed in Table 1. Databases searched included MEDLINE, EMBASE, PsycINFO and Cochrane Centrale Register of Controlled Trials.

## Code availability

No custom code was used in this study.
