# Supplementary material for: A systematic review of pharmacogenetic testing to guide antipsychotic treatment
Source: Nat Ment Health. 2024 Apr 17;2(5):616–26. doi: 10.1038/s44220-024-00240-2 (PMC11088993; doi:10.1038/s44220-024-00240-2)
Supplement: Supplementary file 1 — Supplementary Tables 1–4. [file 44220_2024_240_MOESM1_ESM.pdf]

nature mental health

Analysis

https://doi.org/10.1038/s44220-024-00240-2

A systematic review of pharmacogenetic testing to guide antipsychotic treatment

In the format provided by the authors and unedited

Supplementary Table 1. Characteristics of the excluded studies.

Study	Reason for exclusion	Identification method
**Brixner et al., (1)**	Antipsychotics did not constitute the great majority of prescribed medications	Google Scholar
**Ariefdjohan et al., (2)**	No intervention was provided	Citation search
**Chou et al., (3)**	No intervention was provided	Citation search
**Luke et al., (4)**	No intervention was provided	Citation search
**Battersby (5)**	Not yet recruiting/recruitment incomplete	Databases
**Kennedy and Dechairo (6)**	Unable to retrieve	Databases
**Kahn (7)**	No pharmacogenetic test conducted	Databases
**Zhang (8)**	Unable to retrieve	Databases
**Su et al., (9)**	Not yet recruiting/recruitment incomplete	Databases
**Mueller et al., (10)**	Limited medication information	Databases
**Loew (11)**	Limited medication information	Databases
**Tsermpini et al., (12)**	Not yet recruiting/recruitment incomplete	Databases
**Daut et al., (13)**	Limited medication information	Databases
**Cheema et al., (14)**	Limited medication information	Databases
**McCarthy et al., (15)**	Antipsychotics did not constitute the great majority of prescribed medications	Databases
**Winner et al., (16)**	No intervention was provided	Database
**Actrn (17)**	Not yet recruiting/recruitment incomplete	Database
**Laika et al., (18)**	No intervention was provided	Database
**Ruaño et al., (19)**	Antipsychotics did not constitute the great	Database

	majority of prescribed medications	
**Winner et al., (20)**	Antipsychotics did not constitute the great majority of prescribed medications	Database
**Fagerness et al., (21)**	Antipsychotics did not constitute the great majority of prescribed medications	Citation search
**Olson et al., (22)**	Antipsychotics did not constitute the great majority of prescribed medications	Citation search
**Swen et al., (23)**	Antipsychotics did not constitute the great majority of prescribed medications	Database
**Ramsey and Griffin (24)**	Antipsychotics did not constitute the great majority of prescribed medications	Citation search
**Espadaler et al., (25)**	Antipsychotics did not constitute the great majority of prescribed medications	Citation search
**Breaux et al., (26)**	Antipsychotics did not constitute the great majority of prescribed medications	Database
**Tanner et al., (27)**	Antipsychotics did not constitute the great majority of prescribed medications	Database
**Rodieux et al., (28)**	Limited medication information	Database

Supplementary Table 2. Results from the Downs and Black checklist. UTD, unable to determine.

Reporting	Jürgens et al., (29)	Arranz et al., (30)	Carrascal-Laso et al., (31)	Walden et al., (32)	Arranz et al., (33)	Kang et al., (34)
1. Is the objective of the study clear? (Yes/No)	1	1	1	1	1	1
2. Are the main outcomes clearly described in the Introduction or Methods? (Yes/No)	1	1	1	1	1	1
3. Are the characteristics of the patients included in the study clearly described? (Yes/No)	1	1	1	0	1	1
4. Are the interventions clearly described? (Yes/No)	1	1	1	1	1	1
5. Are the distributions of principal confounders in each group of subjects clearly described? (Yes/Partially/No)	2	2	2	2	2	2
6. Are the main findings of the study clearly described? (Yes/No)	1	1	1	1	1	1
7. Does the study estimate random variability in data for main outcomes? (Yes/No)	1	1	1	1	1	1
8. Have characteristics of patients lost to follow-up been described? (Yes/No)	1	0	0	1	0	1
9. Have actual probability values been reported for the main outcomes except probability < 0.001? (Yes/No)	1	1	0	1	1	1
10. Is the source of funding stated? (Yes/No)	1	1	1	1	1	1
**External validity**						

11. Were subjects who were asked to participate in the study representative of the entire population recruited? (Yes/UTD/No)	1	UTD	UTD	UTD	UTD	UTD
12. Were those subjects who were prepared to participate representative of the recruited population? (Yes/UTD/No)	1	UTD	UTD	UTD	UTD	UTD
13. Were staff, places, and facilities where patients were treated representative of treatment most received? (Yes/UTD/No)	1	1	1	0	0	1
**Internal validity - bias**						
14. Was an attempt made to blind study subjects to the intervention? (Yes/UTD/No)	0	1	0	0	0	1
15. Was an attempt made to blind those measuring the main outcomes? (Yes/UTD/No)	1	1	0	0	0	1
16. If any of the results of the study were based on data dredging was this made clear? (Yes/UTD/No)	1	1	1	1	1	1
17. Was the time period between intervention and outcome the same for intervention and control groups or adjusted for? (Yes/UTD/No)	1	1	1	1	1	1
18. Were the statistical tests used to assess main outcomes appropriate? (Yes/Unclear/No)	1	1	1	1	1	1
19. Were main outcome measures used accurate? (valid and reliable) (Yes/UTD/No)	1	1	1	1	1	1
**Internal validity - confounding**						

20. Were patients in different intervention groups recruited from the same population? (Yes/UTD/No)	1	1	1	1	1	1
21. Were study subjects in different intervention groups recruited over the same period of time? (Yes/UTD/No)	1	UTD	1	UTD	UTD	1
22. Were study subjects randomized to intervention groups? (Yes/UTD/No)	1	1	0	0	0	1
23. Was the randomized intervention assignment concealed from patients and staff until recruitment was complete? (Yes/UTD/No)	0	1	0	0	0	1
24. Was there adequate adjustment for confounding in the analyses from which main findings were drawn? (Yes/UTD/No)	1	1	0	0	0	0
25. Were losses of patients to follow-up taken into account? (Yes/UTD/No)	1	UTD	UTD	1	UTD	1
**Power**						
26. Did the study conduct a power calculation? (Yes/No)	1	1	1	0	0	1
**Total (/27)**	25	22	17	15	15	24

Supplementary Table 3. Results from the Consolidated Health Economic Evaluation Reporting Standards (CHEERS) 2022 checklist. N/A, not applicable.

**Reporting**	**Herbild et al., (35)**	**Carrascal-Laso et al., (36)**	**Perlis et al., (37)**	**Ninomiya et al., (38)**	**Girardin et al., (39)**	**Kurylev et al., (40)**	**Rejon-Parrilla et al., (41)**
**Title**							
1. Title	1	1	1	1	1	1	1
**Abstract**							
2. Abstract	1	1	1	1	1	1	1
**Introduction**							
3. Background and objectives	1	1	1	1	1	1	1
**Methods**							
4. Health economic analysis plan	0	0	0	0	0	0	0
5. Study population	1	1	1	1	1	1	1
6. Setting and location	1	1	0	1	1	1	0
7. Comparators	1	0	1	1	1	0	1
8. Perspective	0	0	0	1	1	0	0
9. Time horizon	0	0	0	1	1	0	1
10. Discount rate	0	0	0	1	0	0	1
11. Selection of outcomes	N/A	N/A	1	1	1	N/A	1
12. Measurement of outcomes	N/A	N/A	1	1	1	N/A	1

13. Valuation of outcomes	N/A	N/A	1	0	0	N/A	1
14. Measurement and valuation of resources and costs	1	1	1	1	1	1	1
15. Currency, price date, and conversion	1	1	1	0	0	0	0
16. Rationale and description of model	N/A	N/A	1	1	1	1	1
17. Analytics and assumptions	1	0	0	1	1	0	1
18. Characterizing heterogeneity	1	0	0	0	0	0	0
19. Characterising distributional effects	0	0	0	0	0	0	0
20. Characterizing uncertainty	1	0	1	1	1	1	1
21. Approach to engagement with patients and others affected by the study	0	0	0	0	0	0	0
**Results**							
22. Study parameters	N/A	N/A	1	1	1	1	1
23. Summary of main results	1	1	1	1	1	1	1
24. Effect of uncertainty	1	0	1	1	1	1	1
25. Effect of engagement with patients and others affected by the study	0	0	0	0	0	0	0
**Discussion**							
26. Study findings, limitations, generalizability, and current knowledge	1	1	1	1	1	0	1
**Other relevant information**							
27. Source of funding	1	0	0	1	1	0	1

28. Conflicts of interest	1	1	0	1	1	1	1
**Total**	70%	43%	57%	75%	71%	48%	71%

Supplementary Table 4. A summary of the GRADE ranking for each outcome.

Author	Studies	Risk of bias	Inconsistency	Indirect evidence	Imprecision	Final ranking
Clinical outcomes						
Adverse drug reactions	3	Minimal concerns	Minimal concerns	Serious	Serious	Low
Symptom severity	4	Serious	Serious	Serious	Serious	Very low
Clinicians' opinions	1	Serious	Serious	Serious	Very serious	Very low
Hospitalisation	3	Serious	Serious	Serious	Serious	Very low
Medication prescribing	2	Very serious	Minimal concerns	Minimal concerns	Serious	Very low
Economic outcomes						
Overall costs	2	Serious	Minimal concerns	Minimal concerns	Serious	Low
Inpatient costs	3	Serious	Minimal concerns	Minimal concerns	Serious	Low
Non-inpatient costs	2	Very serious	Serious	Minimal concerns	Serious	Low
Incremental cost-effectiveness ratio	4	Serious	Serious	Minimal concerns	Serious	Low

REFERENCES

1. Brixner D, Biltaji E, Bress A, Unni S, Ye X, Mamiya T, et al. The effect of pharmacogenetic profiling with a clinical decision support tool on healthcare resource utilization and estimated costs in the elderly exposed to polypharmacy. Journal of Medical Economics. 2016;19(3):213-28.
2. Ariefdjohan M, Lee YM, Stutzman DL, Lenoue S, Wamboldt MZ. The Utility of Pharmacogenetic-Guided Psychotropic Medication Selection for Pediatric Patients: A Retrospective Study. Pediatric Reports. 2021;13(3):421-33.
3. Chou WH, Yan FX, de Leon J, Barnhill J, Rogers T, Cronin M, et al. Extension of a pilot study: impact from the cytochrome P450 2D6 polymorphism on outcome and costs associated with severe mental illness. J Clin Psychopharmacol. 2000;20(2):246-51.
4. Luke MJ, Krupetsky N, Liu H, Korenvain C, Crown N, Toenjes S, et al. Pharmacists as Personalized Medicine Experts (PRIME): Experiences Implementing Pharmacist-Led Pharmacogenomic Testing in Primary Care Practices. Pharmacy. 2021;9(4):201.
5. Battersby M. Impact of Pharmacogenetic Testing on Cost Effectiveness in Mental Illness ANZCTR Identifier: ACTRN12621001222831 2021 [Available from: https://anzctr.org.au/Trial/Registration/TrialReview.aspx?id=381888&showOriginal=true&isReview=true.
6. Kennedy JL, Dechairo B. Pharmacogenomic Decision Support With GeneSight Psychotropic to Guide the Treatment With Antipsychotics ClinicalTrials.gov Identifier: NCT025731682020 [updated 19/11/20]. Available from: https://clinicaltrials.gov/ct2/show/NCT02573168.
7. Kahn R. Optimization of Treatment and Management of Schizophrenia in Europe (OPTIMISE) ClinicalTrials.gov Identifier: NCT012481952016 [updated 15/05/2018]. Available from: https://clinicaltrials.gov/ct2/show/NCT01248195.
8. Zhang J. Prospective Pharmacogenetic Testing and Clinical Outcomes in Patients With Early-Phase Psychosis ClinicalTrials.gov Identifier: NCT025660572017 [updated 24/01/18]. Available from: https://clinicaltrials.gov/ct2/show/NCT02566057.
9. Su Y, Yu H, Wang Z, Liu S, Zhao L, Fu Y, et al. Protocol for a pharmacogenomic study on individualised antipsychotic drug treatment for patients with schizophrenia. BJPsych Open. 2021;7(4).
10. Mueller DJ, Tiwari AK, Soibel A, Likhodi O, MacKenzie B, Richter P, Kennedy JL. CYP2D6 AND CYP2C19 GENE TESTING IN PATIENTS TREATED WITH ANTIPSYCHOTIC AND ANTIDEPRESSANT MEDICATION. Schizophrenia Bulletin. 2011;37(S1):89-90.
11. Loew TH. Pharmacogenetics in psychosomatics - Where is the profit? Journal of Psychosomatic Research. 2019;121.
12. Tsermpini EE, Skokou M, Ferentinos P, Georgila E, Gourzis P, Assimakopoulos K, Patrinos GP. Clinical implementation of preemptive pharmacogenomics in psychiatry: The "PREPARE" study. Psychiatriki. 2020;31(4):341-51.
13. Daut R, Yu K, Li J, Burns L, Brown K, Pollack M, et al. Pharmacogenomic Testing to Inform Prescribing in Patients with Behavioral and Psychiatric Symptoms of Dementia (BPSD): Results from Two Small, Randomized, Controlled Trials. The American Journal of Geriatric Psychiatry. 2021;29(4):S113-S5.
14. Cheema S, Shahmirian A, Zai G, Tiwari A, Herbert D, Braganza N, et al. SU97EFFECT OF PHARMACOGENETICS-GUIDED ANTIDEPRESSANT TREATMENT ON SUICIDAL IDEATION. European Neuropsychopharmacology. 2019;29:S1317.
15. McCarthy L, Sproule B, Crown N, Piquette-Miller MM, Daniel. Pharmacist-led pharmacogenomics services in primary care: Preliminary findings from the PRIME study. Canadian Pharmacists Journal. 2011;150(4):1-327.
16. Winner J, Allen JD, Anthony Altar C, Spahic-Mihajlovic A. Psychiatric pharmacogenomics predicts health resource utilization of outpatients with anxiety and depression. Translational Psychiatry. 2013;3(3):e242-e.

17. Actrn. Impact of Pharmacogenetic Testing on Cost Effectiveness in Mental Illness. https://trialsearchwhoint/Trial2aspx?TrialID=ACTRN12621001222831. 2021.

18. Laika B, Leucht S, Heres S, Steimer W. Intermediate metabolizer: increased side effects in psychoactive drug therapy. The key to cost-effectiveness of pretreatment CYP2D6 screening? The Pharmacogenomics Journal. 2009;9(6):395-403.

19. Ruaño G, Robinson S, Holford T, Mehendru R, Baker S, Tortora J, Goethe JW. Results of the CYP-GUIDES randomized controlled trial: Total cohort and primary endpoints. Contemp Clin Trials. 2020;89:105910.

20. Winner JG, Carhart JM, Altar CA, Goldfarb S, Allen JD, Lavezzari G, et al. Combinatorial pharmacogenomic guidance for psychiatric medications reduces overall pharmacy costs in a 1 year prospective evaluation. Current Medical Research and Opinion. 2015;31(9):1633-43.

21. Fagerness J, Fonseca E, Hess GP, Scott R, Gardner KR, Koffler M, et al. Pharmacogenetic-guided psychiatric intervention associated with increased adherence and cost savings. Am J Manag Care. 2014;20(5):e146-56.

22. Olson MC, Maciel A, Gariepy JF, Cullors A, Saldivar J-S, Taylor D, et al. Clinical Impact of Pharmacogenetic-Guided Treatment for Patients Exhibiting Neuropsychiatric Disorders. The Primary Care Companion For CNS Disorders. 2017;19(02).

23. Swen JJ, van der Wouden CH, Manson LE, Abdullah-Koolmees H, Blagec K, Blagus T, et al. A 12-gene pharmacogenetic panel to prevent adverse drug reactions: an open-label, multicentre, controlled, cluster-randomised crossover implementation study. Lancet. 2023;401(10374):347-56.

24. Ramsey T, Griffin E. Use of Pharmacogenetic Testing in Routine Clinical Practice Improves Outcomes for Psychiatry Patients. Journal of Psychiatry. 2016;19(4).

25. Espadaler J, Tuson M, Lopez-Ibor JM, Lopez-Ibor F, Lopez-Ibor MI. Pharmacogenetic testing for the guidance of psychiatric treatment: a multicenter retrospective analysis. CNS Spectrums. 2017;22(4):315-24.

26. Breaux S, Desrosiers FAD, Neira M, Sinha S, Nislow C. Pharmacogenomics at the Point of Care: A Community Pharmacy Project in British Columbia. Journal of Personalized Medicine. 2020;11(1):11.

27. Tanner J-A, Brown LC, Yu K, Li J, Dechairo BM. <p>Canadian Medication Cost Savings Associated with Combinatorial Pharmacogenomic Guidance for Psychiatric Medications</p>. ClinicoEconomics and Outcomes Research. 2019;Volume 11:779-87.

28. Rodieux F, Daali Y, Rollason V, Samer CF, Ing Lorenzini K. Practice of CYP450 genotyping and phenotyping in children in a real-life setting. Frontiers in Pharmacology. 2023;14.

29. Jürgens G, Andersen SE, Rasmussen HB, Werge T, Jensen HD, Kaas-Hansen BS, Nordentoft M. Effect of Routine Cytochrome P450 2D6 and 2C19 Genotyping on Antipsychotic Drug Persistence in Patients With Schizophrenia. JAMA Network Open. 2020;3(12):e2027909.

30. Arranz MJ, Gonzalez-Rodriguez A, Perez-Blanco J, Penadés R, Gutierrez B, Ibañez L, et al. A pharmacogenetic intervention for the improvement of the safety profile of antipsychotic treatments. Translational Psychiatry. 2019;9(1).

31. Carrascal-Laso L, Franco-Martín MÁ, García-Berrocal MB, Marcos-Vadillo E, Sánchez-Iglesias S, Lorenzo C, et al. Application of a Pharmacogenetics-Based Precision Medicine Model (5SPM) to Psychotic Patients That Presented Poor Response to Neuroleptic Therapy. Journal of Personalized Medicine. 2020;10(4):289.

32. Walden LM, Brandl EJ, Tiwari AK, Cheema S, Freeman N, Braganza N, et al. Genetic testing for CYP2D6 and CYP2C19 suggests improved outcome for antidepressant and antipsychotic medication. Psychiatry Res. 2019;279:111-5.

33. Arranz MJ, Salazar J, Bote V, Artigas-Baleri A, Serra-Llovich A, Triviño E, et al. Pharmacogenetic Interventions Improve the Clinical Outcome of Treatment-Resistant Autistic Spectrum Disorder Sufferers. Pharmaceutics. 2022;14(5):999.
34. Kang Z, Qin Y, Sun Y, Lu Z, Sun Y, Chen H, et al. Multigenetic Pharmacogenomics–Guided Treatment vs Treatment As Usual Among Hospitalized Men With Schizophrenia. JAMA Network Open. 2023;6(10):e2335518.
35. Herbild L, Andersen SE, Werge T, Rasmussen HB, Jürgens G. Does Pharmacogenetic Testing for CYP450 2D6 and 2C19 Among Patients with Diagnoses within the Schizophrenic Spectrum Reduce Treatment Costs? Basic & Clinical Pharmacology & Toxicology. 2013;113(4):266-72.
36. Carrascal-Laso L, Franco-Martín MÁ, Marcos-Vadillo E, Ramos-Gallego I, García-Berrocal B, Mayor-Toranzo E, et al. Economic Impact of the Application of a Precision Medicine Model (5SPM) on Psychotic Patients. Pharmacogenomics and Personalized Medicine. 2021;Volume 14:1015-25.
37. Perlis RH, Ganz DA, Avorn J, Schneeweiss S, Glynn RJ, Smoller JW, Wang PS. Pharmacogenetic testing in the clinical management of schizophrenia: a decision-analytic model. J Clin Psychopharmacol. 2005;25(5):427-34.
38. Ninomiya K, Saito T, Ikeda M, Iwata N, Girardin FR. Pharmacogenomic-guided clozapine administration based on HLA-DQB1, HLA-B and SLCO1B3-SLCO1B7 variants: an effectiveness and cost-effectiveness analysis. Front Pharmacol. 2022;13:1016669.
39. Girardin FR, Poncet A, Perrier A, Vernaz N, Pletscher M, F. Samer C, et al. Cost-effectiveness of HLA-DQB1/HLA-B pharmacogenetic-guided treatment and blood monitoring in US patients taking clozapine. The Pharmacogenomics Journal. 2019;19(2):211-8.
40. Kurylev AA, Andreev BV, Kolbin AS, Limankin OV. CYP2D6 genotyping in the daily routine of a psychiatric hospital – pharmacoeconomic evaluation. PHARMACOECONOMICS Modern pharmacoeconomics and pharmacoepidemiology. 2018;11(1):19-26.
41. Rejon-Parrilla JC, Nuijten M, Redekop WK, Gaultney JG. Economic evaluation of the use of a pharmacogenetic diagnostic test in schizophrenia. Health Policy and Technology. 2014;3(4):314-24.
